# Listening to the Patient’s Voice: A Quantitative Study on Patient-Centredness in Diabetes Care in Palestinian Public Primary Care Services

**DOI:** 10.3390/healthcare14121747

**Published:** 2026-06-17

**Authors:** Hiba Ziad AbuZayyad, Shahenaz Najjar

**Affiliations:** 1 Faculty of Public Health, Al-Quds University, Jerusalem P.O. Box 51000, Palestine; 2 Department of Computer Science, KU Leuven, B-3001 Leuven, Belgium; 3 Augment, Imec Research Group at KU Leuven, B-3001 Leuven, Belgium

**Keywords:** patient-centred care, shared decision-making, primary healthcare, quality healthcare, type 2 diabetes, Palestine

## Abstract

**Highlights:**

**What are the main findings?**

- Perceptions of diabetic patients regarding patient-centred care (PCC) were moderate in government primary healthcare settings.
- A significant variation in PCC evaluation was observed across different governorates and types of residences (village, camp, urban).

**What are the implications of the main findings?**

- Targeted quality-enhancement initiatives are essential for strengthening PCC within diabetes services.
- Further exploration of the relationship between regional disparities and perceptions of PCC is recommended.

**Abstract:**

**Background/Objectives:** Despite the growing burden of type 2 diabetes in Palestine and the central role of patient-centred care (PCC) in high-quality primary healthcare, evidence on PCC from the perspective of people with diabetes remains limited. This study aimed to assess patient-centredness in governmental primary healthcare centres in the West Bank from the perspective of adults with diabetes. **Methods:** A cross-sectional study was implemented in three primary healthcare directorates covering north, south, and central West Bank (WB). The perspectives of patients with type 2 diabetes mellitus (DM) on patient-centredness were investigated using an Arabic-translated version of the PPPC-R questionnaire. A total of 450 eligible patients were approached using non-probability convenience and quota sampling across the three directorates between August and September 2025. We used R (version 4.5.1) for the analysis. **Results:** A total of 417 patients completed the questionnaire (response rate 91%). Participants were (50.4%) women and (49.6%) men, with a mean age of 54.6 years (SD = 12.9). Participants reported moderate overall PCC perceptions (M = 2.82, SD = 0.50), with the highest mean scores for Enhancing the Clinician–Patient Relationship (M = 2.90, SD = 0.52), followed by Understanding the Whole Person (M = 2.77, SD = 0.56) and Finding Common Ground (M = 2.71, SD = 0.71). After adjustment for sociodemographic variables in multivariable analysis HC3-robust regression models, no predictor remained independently significant, and the models explained only a modest share of variance (R^2^ ≈ 0.03–0.06). **Conclusion:** Perceived patient-centredness of diabetes care in governmental PHC clinics in the West Bank was moderate and varied by geographic and contextual factors. Findings suggest a need for targeted quality improvement initiatives to strengthen PCC in diabetes services and to expand the research to other governorates to obtain a clearer picture of the regional disparities within the Palestinian PHC system.

## 1. Introduction

Type 2 Diabetes Mellitus poses a serious public health issue worldwide, with complications that can lead to premature death and disability [1]. A high proportion of people with DM (80.6%, 432.7 million) live in low- and middle-income countries (LMICs), and the highest comparative prevalence of DM was in the Middle Eastern and North African (MENA) Region (18.1%) [2]. Palestine showed similar results with a rise in the prevalence of DM among individuals aged 40 to 69 to 20.8% in 2022, and a higher prevalence in females than males (24.3% and 17.3%, respectively) [3].

The presence of diabetes-related complications and multimorbidity underscores the need for care approaches that account for patients’ individual needs, priorities, and lived experiences. Patients’ perceptions are influenced by their beliefs, values, cultural background, age, education level, health literacy, socioeconomic status, and past healthcare experiences [4]. Recognizing and responding to these individual differences is central to the delivery of high-quality care.

In response to this complexity, Patient-centred care (PCC) has emerged as a key framework for delivering high-quality care. PCC is defined by the Institute of Medicine as care that is respectful of and responsive to individual patient preferences, needs, and values, ensuring that patient values guide all clinical decisions [5]. Mead and Bower’s provided a more practical conceptualization of PCC, combining both the patient and the provider perspectives and roles as functions to be fulfilled during service provision; including therapeutic patient–provider relationship building, bio-psychosocial care provision, individualizing the patient as a unique person, sharing power and responsibility and being realistic of the doctor as a person [6].

Various studies evaluated PCC in different settings. For example, a qualitative study in Germany explored the perspectives of 20 chronic patients on the most important aspect of PCC. The findings identified three major themes: timely and appropriate access to care; healthcare providers’ competence, empathy and the feeling of being taken seriously; and individualized consideration of each patient’s situation [7]. In Saudi Arabia, a cross-sectional study of patients with diabetes showed that the perceived level of PCC was significantly related to patients’ satisfaction [8]. Similarly, evidence from low- and middle-income countries indicates that patients’ perceptions of PCC are shaped by factors such as length of stay, intimacy with providers, privacy during care and easy access to service, as revealed by a facility-based comparative cross-sectional study in Ethiopian public and private hospitals [9]. In Malawi, a qualitative study involving DM patients, healthcare providers and policy makers, conceptualized PCC as encompassing both interpersonal and technical dimensions, highlighting the importance of meeting individual needs and expectations, fostering relationships and patient involvement, facilitating information sharing, and ensuring timely access to medications and services [10].

In Palestine, PCC in primary healthcare (PHC) was largely driven by essential drug availability and respectful provider behaviour, with confidentiality and provider–client interactions further shaping patient perceptions [11]. In hospital settings, patient experience was rated as moderate overall and varied significantly according to sociodemographic and institutional factors [12]. Despite these insights, patient-centredness remains an under-investigated dimension of healthcare quality in Palestine. This gap is particularly concerning given the substantial burden of type 2 diabetes mellitus and the critical role of high-quality PHC in effective disease management [13]. To date, the perspectives and experiences of people with diabetes within PHC settings have not been adequately explored. Existing research has focused predominantly on diabetes risk factors, clinical outcomes, and adherence to clinical protocols, with limited attention to patient experience and the human dimension of care.

While previous studies have examined patient experience in hospital and PHC settings, none have specifically assessed PCC among adults with diabetes attending governmental PHC clinics in the West Bank, which constitute the core setting for the national diabetes programme. Apart from one study conducted in the Gaza Strip that evaluated PCC as a general quality domain in PHC centres [11], no diabetes-specific or West Bank–focused investigations have been undertaken. This highlights a clear gap in context-specific evidence on patient-centred care for diabetes within governmental primary healthcare in Palestine.

Addressing this gap, this study investigates the perspectives of adults with type 2 diabetes on patient-centred care in governmental PHC centres in the WB and compares findings across PHC directorates. By generating baseline evidence on patients’ perceptions of PCC, this study aims to inform quality improvement initiatives in diabetes care within the Palestinian PHC system. Specifically, we addressed two questions: how do adults with type 2 diabetes perceive patient-centred care across its three domains; and whether these perceptions differ significantly by sociodemographic and geographic characteristics.

## 2. Materials and Methods

### 2.1. Study Setting

PHC services are provided in the WB through 14 directorates, distributed across the WB’s governorates. In this study, three governmental primary healthcare directorates from the south, north, and central regions of the WB were selected based on the number of DM patients they serve [14]. Throughout this manuscript, “PHC services” refer to these governmental primary healthcare clinics; the two terms are used interchangeably.

### 2.2. Sampling

A non-probability sampling approach combining convenience and quota sampling was used to overcome movement restrictions between West Bank governorates and reduced working days in Ministry of Health facilities during the study period, following medical syndicate actions [15]. To mitigate potential selection bias and enhance representativeness across key participant characteristics, a quota sampling approach was applied in parallel [15,16]. This approach is consistent with sampling strategies used in previous studies among adults with type 2 diabetes in similar settings [17,18].

### 2.3. Participants and Procedure

A cross-sectional study was conducted among 417 adult patients attending governmental primary healthcare clinics in Palestine. Informed consent was obtained from participants after explaining the research objectives. Eligible participants were aged 18 years or older, had a confirmed diagnosis of type 2 diabetes mellitus, were registered at a governmental primary healthcare clinic, and had attended the clinic at least once in the preceding three months. Patients with type 1 diabetes mellitus or gestational diabetes were excluded. Additional exclusion criteria included first-time clinic visitors, individuals registered for less than one year at the clinic, and those with severe cognitive impairment. Severe cognitive impairment was not assessed with a formal screening instrument; it was identified on the basis of a documented diagnosis or an inability to complete the interview, and this is acknowledged as a limitation.

### 2.4. Data Collection Instrument

Data were collected using an Arabic-translated version of the Revised Patient Perception of Patient-Centredness questionnaire (PPPC-R). The instrument was originally developed to measure three theorized domains of patient-centred care, with items measured on ordered categorical response scales ranging from three to five points [19,20]. The PPPCR-r was adopted to suit the Palestinian context and the study’s population.

The questionnaire was translated into Arabic by a certified legal translator and subsequently back-translated by three independent experts. The back-translated versions were compared to the original PPPC-R to ensure conceptual and semantic equivalence. The finalized Arabic version was piloted with 50 participants to assess clarity, comprehensibility, and suitability for data collection.

Following validation and contextual adaptation, data were collected by trained independent data collectors with prior experience in health research. Data collectors received standardized training on the concept of PCC, the study objectives, and the administration of the questionnaire. Each item was reviewed in detail to ensure consistent explanation using simple, non-technical language, thereby supporting participant comprehension across different educational and socioeconomic backgrounds.

### 2.5. Statistical Analysis

All statistical analyses were conducted using R version 4.5.1. The psychometric properties of the instrument were evaluated prior to use through exploratory factor analysis (EFA) followed by confirmatory factor analysis (CFA). The lavaan package was used for confirmatory factor analysis (CFA), while psych was employed for exploratory factor analysis (EFA) and reliability estimation. The dimensional structure and psychometric properties of the PPPC-R were examined using EFA on a polychoric correlation matrix with weighted least squares (WLS) extraction and geomin-Q oblique rotation, appropriate for ordered categorical indicators and correlated factors [21]. The final EFA supported a refined three-factor, 17-item structure; EFA fit indices and loadings are reported in the Results. CFA was estimated using WLSMV with θ-parameterisation [22]. Model adequacy was evaluated using CFI, TLI, RMSEA, and SRMR, interpreted as descriptive evidence rather than strict pass/fail thresholds [23,24]. Convergent evidence was evaluated via standardized loadings, and internal consistency was assessed using McDonald’s ω.

All items employed ordered response categories ranging from three to five points, describing increasing levels of agreement or frequency (for example, from “not at all” to “to a great extent” or from “strongly disagree” to “strongly agree”). For the sake of interpretability and comparability with previous PPPC-R applications, all substantive analyses retained the original item coding. One item was originally scored on a five-point scale rather than four points. As a sensitivity check, this item was linearly rescaled to a four-point metric and the factor scores were recomputed; the rescaled and original scores correlated almost perfectly (r ≈ 0.9998) with negligible change in means or rank ordering, so the original coding was retained. For inferential analyses, age was analyzed using OLS regression with HC3 heteroskedasticity-consistent standard errors. Categorical predictors were first tested bivariately using Welch’s *t*-tests (two groups) or Welch’s ANOVA (three or more groups) with Satterthwaite-adjusted degrees of freedom; where Welch’s ANOVA was significant, Games–Howell post hoc tests with Holm adjustment for multiple comparisons were used, and non-parametric Kruskal–Wallis tests with Dunn–Holm comparisons were run as sensitivity analyses. The significance level was set at α = 0.05 (two-tailed) throughout.

Separate multivariable linear regression models with HC3-robust standard errors and HC3-adjusted Type-III omnibus tests were then fitted for each factor to estimate associations after mutual adjustment for all covariates. Because the camp subgroups were very small (origin *n* = 9; residence *n* = 10), origin region and residence type were collapsed to two categories for the multivariable models (camp combined with urban as the reference), whereas the full three-category form was retained for the bivariate analyses.

Robust regression analyses and diagnostic tests were performed using sandwich, lmtest, and Type III analysis of variance. Model diagnostics were conducted using car. Relative importance analyses were carried out using relaimpo, and data management and visualization were performed using the tidyverse suite. This analytical framework enabled a transparent and fully reproducible workflow from raw data processing to final statistical modelling.

## 3. Results

### 3.1. Sample Characteristics

The sample was nearly evenly distributed by gender (50.4% female, 49.6% male) and represented three governorates: Hebron (37.4%), Ramallah (32.4%), and Nablus (30.2%). The mean age of participants was 54.6 years (SD = 12.9), with a median age of 56 years, indicating a middle-aged to older adult population. The dataset contained no missing values across all variables, enabling a complete-case analysis (N = 417). A detailed descriptive analysis is presented in Table 1.

### 3.2. Construct Validity

The results of EFA and CFA to validate the Arabic version of the PPPC-R showed a 17-item solution for the final EFA. Preliminary analyses indicated that the item “To what extent did you and the provider discuss your respective roles?” exhibited problematic cross-loadings and high residual correlations. Therefore, the item was removed in order to achieve a cleaner and more interpretable factor structure. Two items originally theorized to load on the “Common Ground” factor (F3)—“To what extent did your provider explain treatment?” and “To what extent did the provider explore how manageable this treatment would be for you?”—demonstrated primary loadings on the “Whole-Person Understanding” factor (F2) (0.76 and 0.84, respectively), this reassignment was retained for empirical strength, conceptual alignment, theoretical consistency, and psychometric improvement.

The CFA model yielded the following fit indices: CFI = 0.873, TLI = 0.847, RMSEA = 0.129, SRMR = 0.063. These indices provide only partial support for the three-factor structure: the SRMR was acceptable, and the incremental indices were moderate, while the elevated RMSEA and the CFI/TLI values slightly below conventional thresholds suggest the model does not fully capture the data. We therefore interpret the structure as offering partial support for a three-factor model rather than strong confirmatory evidence, possibly reflecting cultural or linguistic instability of the Arabic-adapted scale. All standardized factor loadings were statistically significant (as shown in Table 2), ranging from 0.57 to 0.90, demonstrating strong convergent validity. The factors were highly correlated (φ_12_ = 0.865, φ_13_ = 0.660, φ_23_ = 0.837), indicating that while distinct, the three domains of patient-centredness are closely related in practice. Reliability, assessed with McDonald’s omega, was excellent for all factors (ω > 0.88).

As the final Finding Common Ground factor is based on two items, findings related to shared decision-making are interpreted with some caution and may benefit from further item development in future adaptations.

Finally, three factors were identified:

- Relationship and Communication: This factor encompasses core communication behaviours and the foundational patient–provider relationship, including understanding, listening, trust, and satisfaction.
- Whole-Person Understanding: This factor reflects the provider’s attention to the patient’s personal context, emotions, and beliefs, including compassion, respect, and knowledge of the patient’s life.
- Common Ground: This factor captured the collaborative process of setting treatment goals and encouraging patient agency.

Results of confirmatory factor analysis are shown in Table 2.

### 3.3. Overall Patient Perceptions of PPPC-R Domains

**Figure 1:**
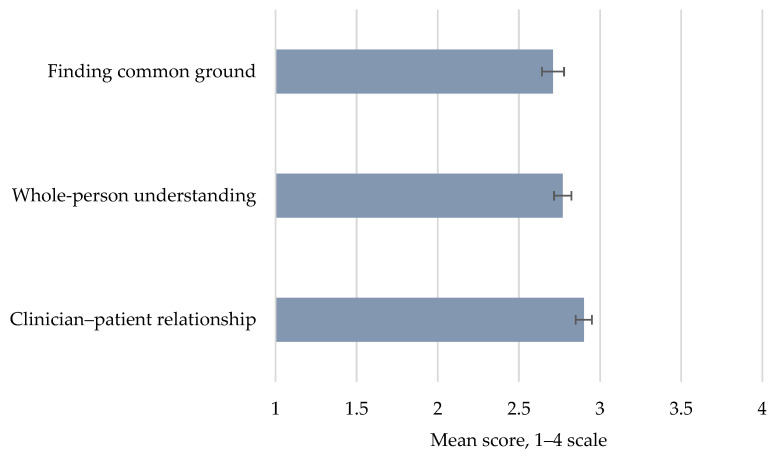
PPPC-R Domain Scores. Means with 95% confidence intervals. Note. Error bars represent 95% confidence intervals around the mean. Higher scores indicate more positive perceptions of patient-centred care. Standard deviations are reported in Table 3.

The overall PPPC-R item-weighted mean score was 2.82 (SD = 0.50), indicating moderately positive perceptions of patient-centredness on the 1–4 scale. Patients reported the highest mean scores for enhancing the clinician–patient relationship (M = 2.90, SD = 0.52), followed by understanding the whole person (M = 2.77, SD = 0.56), and finding common ground (M = 2.71, SD = 0.71), with greater variability observed in finding common ground (Figure 1). The distribution of scores across all three factors indicated generally positive perceptions of PCC, with mean scores falling in the upper half of the response scale range, as shown in Table 3.

### 3.4. PPPC-R Domains’ Means by Demographic Groups

Patient-centredness was measured for the three factors across all demographic subgroups, means and standard deviations. In addition, an overall item-weighted mean across all 17 PPPC-R items was computed to show the overall PCC score. This is presented in Table 4.

Across age groups, Factor 1 means were relatively homogeneous, clustering around 2.81–2.93 for adults aged 30 years and older, with a higher mean among the youngest group (≤29 years; M = 3.38, SD = 0.43). For Factor 2, means ranged from 2.61 to 3.05 across age groups, with the ≤29 group showing the highest mean. Factor 3 showed a similar pattern, with the ≤29 group again reporting the highest mean (M = 3.07, SD = 0.79). Consistent with this pattern, the overall score was also highest in the ≤29 group (M = 3.21, SD = 0.54) and lowest in the 40–49 group (M = 2.73, SD = 0.58).

Gender differences in the descriptive statistics were small in magnitude. Women reported a slightly higher mean on Factor 1 (M = 2.94, SD = 0.54) and slightly lower means on Factors 2 and 3 (M = 2.74, SD = 0.61 and M = 2.65, SD = 0.77, respectively) compared with men (Factor 1: M = 2.86, SD = 0.49; Factor 2: M = 2.80, SD = 0.50; Factor 3: M = 2.76, SD = 0.63). The overall score was nearly identical by gender (female: M = 2.83, SD = 0.53; male: M = 2.82, SD = 0.46). Education-related differences were modest for Factors 1 and 2 but more apparent for Factor 3, where participants with a bachelor’s degree or higher scored higher on Factor 3 than those with secondary education or less, with the diploma group falling between these categories. Overall scores were similar across education levels (M = 2.74–2.84).

Geographical patterns were more evident. For Factor 1, Nablus had the highest mean (M = 3.01, SD = 0.62), Hebron was intermediate (M = 2.90, SD = 0.51), and Ramallah had the lowest mean (M = 2.79, SD = 0.41). For Factor 2, scores were highest in Ramallah (M = 2.84, SD = 0.34) and lower in Hebron and Nablus (both M = 2.74). For Factor 3, scores were highest in Ramallah (M = 2.86, SD = 0.42) and lower in Hebron and Nablus. Across governorates, the overall score showed a relatively narrow range (M = 2.80–2.86). Single participants showed the highest means across all three factors (Factor 1: M = 3.13, SD = 0.62; Factor 2: M = 2.95, SD = 0.64; Factor 3: M = 2.92, SD = 0.82), although the divorced/widowed group was small (*n* = 24). Single participants also had the highest overall mean (M = 3.03, SD = 0.58), whereas married participants reported a lower overall mean (M = 2.80, SD = 0.48). Work status showed lower Factor 1 mean among public employees (M = 2.77, SD = 0.44), lower Factor 2 means among housewives and public employees (M = 2.72, SD = 0.61 and M = 2.74, SD = 0.48, respectively), and lower Factor 3 mean among housewives (M = 2.58, SD = 0.79). Across work status categories, overall scores ranged from 2.76 (public employees; SD = 0.42) to 2.90 (private employees and self-employed; SDs = 0.52 and 0.38, respectively).

For origin region and residence type, a clearer pattern emerged for Factor 3. Participants of village origin (M = 2.83, SD = 0.56) and those currently living in villages (M = 2.83, SD = 0.53) reported higher scores than those of urban origin (M = 2.62, SD = 0.74) and urban residents (M = 2.62, SD = 0.74). The camp groups showed the highest Factor 3 means (camp origin: M = 3.50, SD = 0.79; camp residence: M = 3.65, SD = 0.75), these estimates should be interpreted cautiously because the camp groups were very small (origin: *n* = 9; residence: *n* = 10), which also affects the stability of inferential tests (Table 5, Table 6, Table 7, Table 8, Table 9 and Table 10). For Factor 1, means were essentially similar across urban and village origin/residence (Around 2.88–2.90), while Factor 2 showed a small tendency toward higher scores among village origin/residents (both M = 2.83) compared with urban origin/residents (M = 2.73 and 2.72, respectively). Consistent with the Factor 3 pattern, overall scores were higher in the camp groups (camp origin: M = 3.33, SD = 0.63; camp residence: M = 3.51, SD = 0.58) than in the urban and village groups (both M = 2.79 for urban origin/residence and M = 2.85 for village origin/residence).

### 3.5. Group Differences and Regression Analysis

#### 3.5.1. Bivariate Group Differences

At the bivariate level, residence type and governorate (and origin region for Finding Common Ground) showed the most consistent group differences, whereas age, gender, education, marital status, and work status generally did not. Across all three domains, camp residents reported the highest scores (Table 4).

For Enhancing the Clinician–Patient Relationship, the Welch ANOVA was significant for governorate, *F*(2, 262.52) = 6.11, *p* = 0.003 (Table 5); Post Hoc comparisons indicated that Ramallah scored lower than Nablus (Ramallah–Nablus = −0.22, 95% *CI* [−0.37, −0.07], *p* = 0.002), while the remaining governorate contrasts were not significant (Table 6). Residence type was also significant, *F*(2, 24.22) = 6.91, *p* = 0.004, with urban and village residents scoring lower than camp residents (Urban–Camp = −0.65, 95% *CI* [−1.13, −0.17], *p* = 0.010; Village–Camp = −0.66, 95% *CI* [−1.14, −0.17], *p* = 0.010).

For Understanding the Whole Person, only residence type reached significance, *F*(2, 23.97) = 6.73, *p* = 0.005, again with camp residents scoring higher than both urban residents (Urban–Camp = −0.72, 95% *CI* [−1.33, −0.11], *p* = 0.022) and village residents (Village–Camp = −0.61, 95% *CI* [−1.22, 0.00], *p* = 0.050).

For Finding Common Ground, governorate, origin region, and residence type were all significant. Governorate showed a significant effect, *F*(2, 247.90) = 7.12, *p* = 0.001, with Ramallah scoring higher than both Hebron (Ramallah–Hebron = 0.23, 95% *CI* [0.07, 0.39], *p* = 0.003) and Nablus (Ramallah–Nablus = 0.21, 95% *CI* [0.01, 0.42], *p* = 0.038). Residence type showed the strongest effect, *F*(2, 24.20) = 12.62, *p* < 0.001: camp residents scored higher than both urban (Urban–Camp = −1.03, 95% *CI* [−1.70, −0.37], *p* = 0.004) and village residents (Village–Camp = −0.82, 95% *CI* [−1.49, −0.16], *p* = 0.017), and village residents scored higher than urban residents (Village–Urban = 0.21, 95% *CI* [0.06, 0.36], *p* = 0.004). A parallel pattern emerged for origin region, *F*(2, 21.45) = 8.97, *p* = 0.001, with camp origin scoring higher than urban origin (Urban–Camp = −0.88, 95% *CI* [−1.63, −0.12], *p* = 0.025) and village origin scoring higher than urban origin (Village–Urban = 0.21, 95% *CI* [0.05, 0.36], *p* = 0.006).

**Figure 2:**
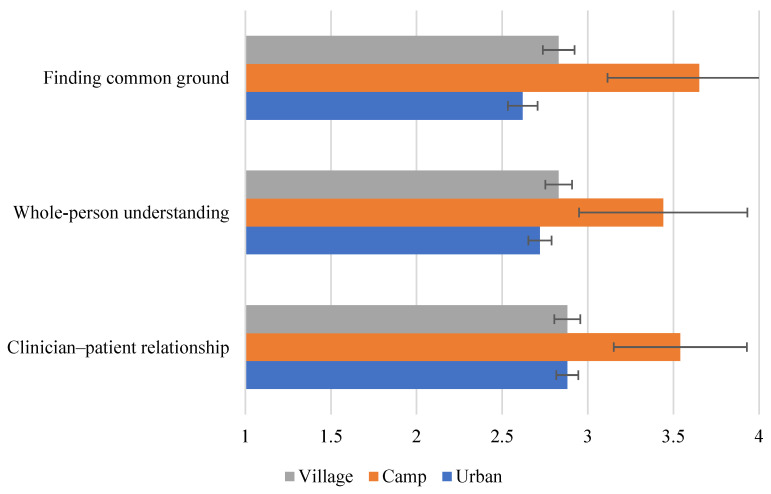
Unadjusted PPPC-R Scores by Residence Type in the Bivariate Analysis. Unadjusted means with 95% confidence intervals. Note. Means are unadjusted and based on the original three-category residence type variable used in the bivariate analyses. Error bars represent 95% confidence intervals. Higher scores indicate more positive perceptions of patient-centred care. Camp estimates should be interpreted cautiously because the camp subgroup was small (*n* = 10). The upper confidence interval for the camp group may extend beyond the theoretical scale maximum because of the small subgroup size.

These bivariate patterns were consistent across the parametric (Welch/Games–Howell) and non-parametric (Kruskal–Wallis/Dunn–Holm) approaches (Table 7). However, the camp subgroups were very small (origin *n* = 9; residence *n* = 10), so the camp contrasts should be interpreted with considerable caution (Figure 2).

#### 3.5.2. Multivariable Regression

**Figure 3:**
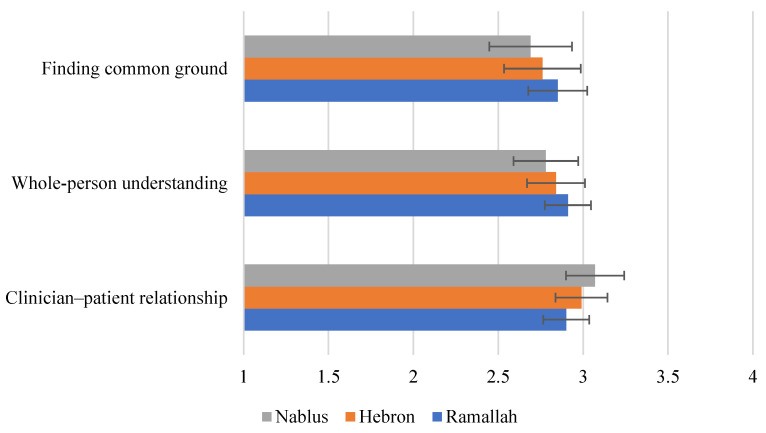
Adjusted PPPC-R Scores by Governorate. Adjusted predicted means with 95% confidence intervals. Note. Adjusted means were estimated from HC3-robust multivariable linear models controlling for age, gender, education, marital status, work status, origin region, and residence type. Error bars represent 95% confidence intervals.

**Figure 4:**
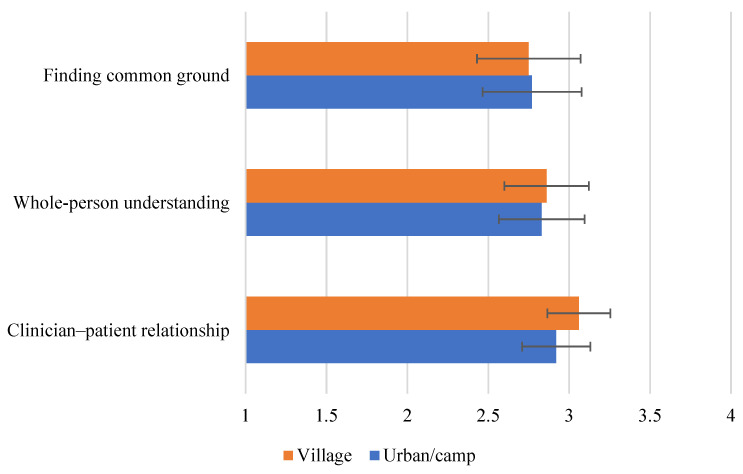
Adjusted PPPC-R Scores by Residence Type. Adjusted predicted means with 95% confidence intervals. Note. Adjusted means were estimated from HC3-robust multivariable linear models controlling for age, gender, education, marital status, work status, origin region, and governorate. Error bars represent 95% confidence intervals around the adjusted predicted means. Higher scores indicate more positive perceptions of patient-centred care. Camp was combined with urban because of the small camp subgroup.

Separate HC3-robust multivariable linear regression models were then fitted for each factor, mutually adjusting for age, gender, education, marital status, work status, governorate, origin region, and residence type, with camp combined with urban as the reference category (Table 8, Table 9 and Table 10). After mutual adjustment, none of the predictors showed a statistically significant HC3-adjusted Type-III omnibus effect for any of the three factors, and the models explained only a modest share of the variance (Factor 1: R^2^ = 0.060; Factor 2: R^2^ = 0.031; Factor 3: R^2^ = 0.058). Thus, clear group differences were apparent in the unadjusted comparisons. Yet these contrasts largely attenuated once the covariates were considered jointly, suggesting that they reflect shared variance and confounding among the contextual variables rather than independent associations (Figure 3). When camp was combined with urban in these models, the adjusted analyses do not directly test camp-specific contrasts (Figure 4); the camp pattern is reported descriptively and in the bivariate analyses only.

### 3.6. Sensitivity Analysis: Original Versus Modified Factor Structure

To assess whether the reassignment of two treatment-related items from Finding Common Ground to Understanding the Whole Person influenced the results, a sensitivity analysis was conducted in which factor scores were recomputed using the original three-domain PPPC-R assignment. The modified and original scores were highly correlated (F1, r = 1.00; F2, r = 0.95; F3, r = 0.90). The substantive findings were unchanged under the original structure: at the bivariate level, Finding Common Ground continued to differ significantly by governorate, residence type, and origin region, while Factors 1 and 2 showed no meaningful residence or origin differences. In the multivariable HC3-robust models, no predictor reached a significant Type-III omnibus effect for any factor (all *p* > 0.05), and model fit was comparably modest (R^2^ = 0.06, 0.02, and 0.07 for Factors 1–3, respectively). These results indicate that the conclusions do not depend on the item reassignment. Full results are reported in the Supplementary Material (Table S1).

## 4. Discussion

This study’s findings showed that the three factors of PCC were highly correlated (φ_12_ = 0.865, φ_13_ = 0.660, φ_23_ = 0.837), suggesting that, in practice, the domains of relationship and communication, whole-person understanding, and common ground frequently co-occur during clinical encounters. This was also shown in a previous study from Gaza, where interactions between healthcare providers and clients, increasing confidentiality and privacy, and ensuring the availability of essential drugs were all correlated with patients’ perceptions of PCC [11]. This is understandable in the cultural context in Palestine, where patients tend to open up to their caregivers and elaborate in their encounters.

Participants in our study reported the highest mean scores for enhancing the clinician–patient relationship (M = 2.90, SD = 0.52), followed by Understanding the Whole Person and Finding Common Ground; however, the means of the three domains were relatively similar (close). These patterns suggest that patients generally perceived their encounters as moderately patient-centred, particularly in the relational and communicative aspects of care. The larger standard deviation for Finding Common Ground indicates that experiences with shared decision-making and goal setting are more heterogeneous than for the other two dimensions and require further exploration; this is consistent with a study from the Netherlands on patients with multimorbidity, which observed that ranges for aspects of PCC delivery extended across almost the full theoretical range, with finding common ground showing the greatest variability [25]. However, this finding needs further exploration since the Arabic version had only two questions validated for this domain, and the need to adjust the Arabic version of the tool to reflect this domain more explicitly may be needed.

There was no significant difference in PCC perception based on age, gender, and education, this is consistent with findings from Trinidad where no statistical differences in the patient perception of patient-centredness were found by gender, employment status, age, ethnicity, religion, marital status, education, income, number of medical problems to discuss, non-communicable disease, number of medications used and general health rating [26]. Similar results were also found in a study from Saudi Arabia, where age and gender were not significantly correlated with perceived PCC or patient satisfaction; however, income and education were substantial factors [8]. Residence type and governorate (and origin region for Factor 3), on the other hand, showed significant differences. Camp and village (rural) residents tended to score higher than urban residents on finding common ground, similarly to a study from Ethiopia that showed poor patient-centred care to be significantly associated with urban residents in public hospitals, but not significantly different in private hospitals [9]. One possible interpretation is that patients in rural and camp areas may have lower expectations of care, whereas urban residents may benefit from greater health literacy and access to health information. However, the camp subgroups were very small (origin *n* = 9; residence *n* = 10), so these specific contrasts should be regarded as exploratory.

Governorate differences were most evident for finding common ground, where Ramallah scored higher than Hebron and Nablus. One possible explanation is Ramallah’s early achievement of PHC accreditation, recent improvements in healthcare infrastructure and services, and broader socioeconomic advantages at the governorate level; however, clinic-level data on accreditation, staffing, consultation length, and medicine availability were not collected, so these explanations remain hypotheses rather than tested mechanisms. It should also be noted that these governorate and residence differences were observed in unadjusted comparisons and did not remain statistically significant after mutual adjustment in the multivariable models, and should therefore not be over-interpreted as independent effects. Nonetheless, the experience of Ramallah in PHC accreditation and infrastructure improvement may be worth examining further as a route toward more uniform standards of care across WB governorates.

In our study, EFA and CFA revealed different factor distributions within the three domains than in the revised-PPPC-R, mainly affecting the domain “finding common grounds”, where one question was omitted from our version, and two other questions reappeared in the second domain “understanding the whole person”. This may be due to contextual factors, including a poor understanding of the patient’s role and greater reliance on the healthcare provider. This is consistent with the original finding in the evaluation of the tool [19], where this question and another one describing the roles of patients in care did not load on the two other domains but were retained in the tool as part of the domain “finding common ground” for their importance in describing PCC. This may also suggest a thematic difference due to language or culture, implying that the Palestinian patients did not perceive the concept of “finding a common ground” with healthcare providers, and rather reflected their experience as recipients of care. Our finding contradicts the findings from Jordan using the same tool, but among the Jordanian population, Q12 (To what extent does your provider know about your family life?) was irrelevant and removed from their tool; Three questions from the first domain loaded better on the second domain [27]. Despite geographical proximity and similar culture, the PPPC-R tool validation showed variable results between Jordan and Palestine.

Understanding the influence of regional and residential context on this interpersonal dimension of patient-centred care requires further investigation, whether by expanding the research for more governorates, or by following an exploratory qualitative approach for the same study population.

## 5. Limitations

Several limitations should be considered when interpreting the findings of this study. First, the use of non-probability sampling may limit the representativeness of the sample and the generalizability of the results. Second, the study was restricted to three directorates, which may not fully reflect the experiences and characteristics of patients in other governorates. Additionally, the cross-sectional design precludes the establishment of causal relationships between the variables studied.

Data were collected using self-reported measures, which may be subject to recall bias and reporting inaccuracies. Conducting data collection within healthcare clinics may also have introduced social desirability bias, whereby participants provided responses they perceived to be more acceptable to healthcare providers.

As for the exclusion criteria, patients with severe cognitive impairment were excluded on the basis of a documented diagnosis or the inability to complete the interview, and not based on a formal screening instrument.

The small sample sizes of certain subgroups, such as camp origin and place of residence, had limited significance in the analysis. Furthermore, modification of the PPPC-r structure may have introduced measurement uncertainty and could affect the comparability of findings with previous studies using the original instrument.

Another limitation is the absence of clinic-level variables, such as staffing levels, waiting times, continuity of care, medication availability, and accreditation status, which may influence patient perceptions and experiences of patient-centred care. Finally, the study did not include important diabetes-related clinical characteristics, such as disease duration, treatment regimen, glycemic control, and diabetes-related complications, which also may have affected the observed associations.

Despite these limitations, several factors support the credibility of the study findings. The study included participants across three major governorates from the north, south and centre of the WB serving the highest number of registered DM Type 2 patients. Standardized data collection procedures and the use of a structured instrument helped enhance the consistency of responses. In addition, many of the observed associations were consistent with findings reported in previous studies, supporting the validity of the results. While causal inferences cannot be made, the identified patterns and relationships offer valuable insights into patient perceptions of care and highlight areas that warrant further investigation through longitudinal and qualitative studies.

## 6. Conclusions

Our findings showed moderate perceptions of PCC in governmental PHC DM clinics, with variations across governorates and place of residence at the bivariate level. This requires further investigation into the underlying factors determining these variations. To understand these variations, we recommend further expanding the study population to include patients with other chronic diseases, not only DM, and expanding to additional governorates. The perceptions among camp and village residents may be further understood through qualitative exploratory research, using focus groups. The study findings highlight the need for improving PCC in PHC centres. This supports the need for qualitative exploratory research to further understand the underlying causes of moderate PCC perceptions and consequently define opportunities for improvement.

A national policy to officially adopt PCC in the provision of governmental healthcare services could be valuable, especially if it supports periodic evaluation of patients’ perceptions to identify opportunity windows for improvement.

## Figures and Tables

**Table 1:** Sample Demographic Characteristics (N = 417).

Characteristic	Category	*n* (%)
Gender	Female	210 (50.4%)
	Male	207 (49.6%)
Governorate	Ramallah	135 (32.4%)
	Hebron	156 (37.4%)
	Nablus	126 (30.2%)
Education Level	Secondary or Less	254 (60.9%)
	Diploma	51 (12.2%)
	Bachelor or Higher	112 (26.9%)
Marital Status	Married	361 (86.6%)
	Single	32 (7.7%)
	Divorced or Widowed	24 (5.8%)
Work Status	Private Employee	46 (11%)
	Daily Worker	35 (8.4%)
	Housewife	156 (37.4%)
	Public Employee	82 (19.7%)
	Self-employed	28 (6.7%)
	Unemployed	70 (16.8%)
Origin Region	Urban	277 (66.4%)
	Village	131 (31.4%)
	Camp	9 (2.2%)
Residence Type	Urban	280 (67.1%)
	Village	127 (30.5%)
	Camp	10 (2.4%)
Age (Years)	Mean (SD)	54.6 (12.9)

**Table 2:** Confirmatory Factor Analysis Results: Standardized Loadings and Reliability (WLSMV; N = 417).

Factor and Item	Standardized Loading
**F1: Enhancing the Clinician–Patient Relationship (ω = 0.93)**	
F1.1: How satisfied were you with the discussion of your problem?	0.74
F1.2: To what extent did you agree with your provider’s opinion about the problem?	0.70
F1.3: How well do you think the provider understood you on that visit?	0.82
F1.4: To what extent was your main problem(s) discussed on that visit?	0.84
F1.6: To what extent did the provider explain this problem to you?	0.76
F1.5: To what extent does your provider really listen to you?	0.82
F1.7: To what extent do you trust your provider?	0.90
F1.8: How much would you say that this provider cares about you as a person?	0.90
**F2: Understanding the Whole Person (ω = 0.93)**	
F2.1: To what extent does your provider know about your family life?	0.57
F2.2: How comfortable are you discussing personal problems related to your health with your provider?	0.74
F2.3: To what extent does your provider show you compassion?	0.86
F2.4: To what extent does your provider consider your thoughts and feelings?	0.80
F2.5: To what extent does your provider respect your beliefs, values and customs?	0.82
F3.1: To what extent did your provider explain treatment?	0.58
F3.2: To what extent did the provider explore how manageable this treatment would be for you?	0.59
**F3: Finding Common Ground (ω = 0.88)**	
F3.4: To what extent did the provider ask about your goals for treatment?	0.88
F3.5: To what extent did the provider encourage you to take the role you wanted in your own care?	0.80

Note. Item codes (F1.1–F3.5) denote the original revised PPPC-R domain assignment [19,20]: F1 = Relationship and Communication, F2 = Whole-Person Understanding, F3 = Common Ground. Two items originally assigned to Common Ground (F3.1, F3.2) loaded primarily on Understanding the Whole Person and were reassigned accordingly. One original Common Ground item (F3.3, “To what extent did you and the provider discuss your respective roles?”) was removed because of cross-loadings and high residual correlations. All loadings *p* < 0.001. ω = McDonald’s omega.

**Table 3:** Descriptive Statistics for PPPC-R Factor Scores (N = 417).

Factor	Mean	SD	Original Range	Observed Range
F1: Enhancing the Clinician–Patient Relationship	2.90	0.52	1–4	1.13–4.00
F2: Understanding the Whole Person	2.77	0.56	1–4	1.00–4.00
F3: Finding Common Ground	2.71	0.71	1–4	1.00–4.00
Overall PPPC-R	2.82	0.50	1–4	1.18–4.00

Note. PPPC-R = Patient Perception of Patient-Centredness–Revised.

**Table 4:** PPPC-R Factor Means by Demographic Characteristics (N = 417).

Variable	Category	*n*	M (SD)			
			F1	F2	F3	Overall
Age group	≤29	22	3.38 (0.43)	3.05 (0.74)	3.07 (0.79)	3.21 (0.54)
	30–39	30	2.81 (0.57)	2.83 (0.62)	2.75 (0.85)	2.81 (0.56)
	40–49	51	2.85 (0.60)	2.61 (0.62)	2.68 (0.79)	2.73 (0.58)
	50–59	140	2.82 (0.46)	2.77 (0.51)	2.68 (0.63)	2.78 (0.45)
	60+	174	2.93 (0.51)	2.77 (0.53)	2.68 (0.70)	2.84 (0.47)
Gender	Female	210	2.94 (0.54)	2.74 (0.61)	2.65 (0.77)	2.83 (0.53)
	Male	207	2.86 (0.49)	2.80 (0.50)	2.76 (0.63)	2.82 (0.46)
Education	Secondary or less	254	2.95 (0.52)	2.78 (0.58)	2.63 (0.75)	2.84 (0.51)
	Bachelor or higher	112	2.85 (0.52)	2.79 (0.51)	2.85 (0.62)	2.83 (0.47)
	Diploma	51	2.76 (0.48)	2.71 (0.53)	2.75 (0.59)	2.74 (0.47)
Governorate	Ramallah	135	2.79 (0.41)	2.84 (0.34)	2.86 (0.42)	2.82 (0.35)
	Hebron	156	2.90 (0.51)	2.74 (0.55)	2.63 (0.73)	2.80 (0.49)
	Nablus	126	3.01 (0.62)	2.74 (0.73)	2.64 (0.88)	2.86 (0.62)
Marital status	Married	361	2.87 (0.50)	2.75 (0.54)	2.70 (0.69)	2.80 (0.48)
	Divorced or widowed	24	3.04 (0.59)	2.84 (0.63)	2.44 (0.78)	2.89 (0.54)
	Single	32	3.13 (0.62)	2.95 (0.64)	2.92 (0.82)	3.03 (0.58)
Work status	Private employee	46	2.93 (0.57)	2.85 (0.57)	2.92 (0.61)	2.90 (0.52)
	Daily worker	35	2.86 (0.44)	2.77 (0.49)	2.69 (0.75)	2.80 (0.45)
	Housewife	156	2.94 (0.53)	2.73 (0.61)	2.58 (0.79)	2.81 (0.53)
	Public employee	82	2.77 (0.44)	2.74 (0.48)	2.78 (0.57)	2.76 (0.42)
	Self-employed	28	2.99 (0.44)	2.81 (0.47)	2.84 (0.51)	2.90 (0.38)
	Unemployed	70	2.91 (0.60)	2.83 (0.59)	2.70 (0.72)	2.86 (0.55)
Origin region	Urban origin	277	2.90 (0.54)	2.73 (0.58)	2.62 (0.74)	2.79 (0.51)
	Camp origin	9	3.38 (0.61)	3.24 (0.74)	3.50 (0.79)	3.33 (0.63)
	Village origin	131	2.88 (0.47)	2.83 (0.47)	2.83 (0.56)	2.85 (0.43)
Residence type	Urban residence	280	2.88 (0.54)	2.72 (0.58)	2.62 (0.74)	2.79 (0.52)
	Camp residence	10	3.54 (0.54)	3.44 (0.69)	3.65 (0.75)	3.51 (0.58)
	Village residence	127	2.88 (0.44)	2.83 (0.45)	2.83 (0.53)	2.85 (0.39)

**Table 5:** Bivariate Omnibus Tests Across the PPPC-R Factors.

		Factor 1		Factor 2		Factor 3	
Predictor	Test (Type)	Statistic (df)	*p*	Statistic (df)	*p*	Statistic (df)	*p*
Age	OLS (HC3) regression	*t*(415) = −1.09	0.278	*t*(415) = −0.33	0.745	*t*(415) = −1.14	0.257
Education level	Welch ANOVA	*F*(2, 131.72) = 2.12	0.131	*F*(2, 132.85) = 0.36	0.783	*F*(2, 141.12) = 2.52	0.089
Gender	Welch t	*t*(411.99) = 1.73	0.085	*t*(401.11) = −1.00	0.320	*t*(401.26) = −1.68	0.094
Governorate	Welch ANOVA	*F*(2, 262.52) = 6.11	0.003	*F*(2, 248.02) = 2.24	0.108	*F*(2, 247.90) = 7.12	0.001
Marital status	Welch ANOVA	*F*(2, 38.75) = 1.98	0.190	*F*(2, 39.00) = 2.48	0.128	*F*(2, 38.97) = 1.42	0.300
Origin region	Welch ANOVA	*F*(2, 21.38) = 2.83	0.081	*F*(2, 21.27) = 3.37	0.053	*F*(2, 21.45) = 8.97	0.001
Residence type	Welch ANOVA	*F*(2, 24.22) = 6.91	0.004	*F*(2, 23.97) = 6.73	0.005	*F*(2, 24.20) = 12.62	<0.001
Work status	Welch ANOVA	*F*(5, 124.73) = 1.87	0.104	*F*(5, 125.86) = 0.60	0.699	*F*(5, 126.98) = 2.37	0.043

Note. Age was tested using simple OLS regression with HC3 heteroskedasticity-consistent standard errors (robust test of the age slope reported as t(415)). Categorical predictors were tested using Welch’s *t* test (2 groups) or Welch’s ANOVA (>2 groups), with Satterthwaite-adjusted degrees of freedom. Age effects (HC3-robust OLS): Factor 1: b = −0.00243 (SEHC3 = 0.00223), t(415) = −1.09, *p* = 0.278, R2 = 0.0037; Factor 2: b = −0.0008 (SEHC3=0.0026), t(415) = −0.33, *p* = 0.745, R2 = 0.0003; Factor 3: b = −0.0033 (SEHC3 = 0.0030), t(415) = −1.14, *p* = 0.257, R2 = 0.0039.

**Table 6:** Games–Howell Post Hoc Comparisons for Significant Predictors for PPPC-R Factors.

Predictor	Comparison	Mean Difference	95% CI	*p_adj*
**Factor 1—Enhancing the Clinician–Patient Relationship**				
Governorate	Ramallah–Nablus	−0.22	[−0.37, −0.07]	0.002
Residence type	Urban–Camp	−0.65	[−1.13, −0.17]	0.010
	Village–Camp	−0.66	[−1.14, −0.17]	0.010
**Factor 2—Understanding the Whole Person**				
Residence type	Urban–Camp	−0.72	[−1.33, −0.11]	0.022
	Village–Camp	−0.61	[−1.22, 0.00]	0.050
	Village–Urban	0.11	[−0.01, 0.23]	0.092
**Factor 3—Finding Common Ground**				
Governorate	Ramallah–Hebron	0.23	[0.07, 0.39]	0.003
	Ramallah–Nablus	0.21	[0.01, 0.42]	0.038
Origin region	Urban origin–Camp origin	−0.88	[−1.63, −0.12]	0.025
	Village origin–Urban origin	0.21	[0.05, 0.36]	0.006
Residence type	Urban–Camp	−1.03	[−1.70, −0.37]	0.004
	Village–Camp	−0.82	[−1.49, −0.16]	0.017
	Village–Urban	0.21	[0.06, 0.36]	0.004
Work status	Private employee–Housewife	0.34	[0.02, 0.66]	0.031

**Table 7:** Non-Parametric Sensitivity Tests for PPPC-R Factors.

Predictor	Kruskal–Wallis H(df)	*p*	Pairwise Comparison *	z	*p_adj*
**Factor 1—Enhancing the Clinician–Patient Relationship**					
Governorate	H(2) = 10.37	0.006	Ramallah vs. Nablus	−3.20	0.004
Marital status	H(3) = 10.89	0.012	Single vs. Married	2.85	0.026
Residence type	H(2) = 11.56	0.003	Urban vs. Camp	−3.32	0.002
			Village vs. Camp	−3.38	0.002
**Factor 2—Understanding the Whole Person**					
Residence type	H(2) = 11.55	0.003	Urban vs. Camp	−3.16	0.005
			Village vs. Camp	−2.58	0.020
			Village vs. Urban	1.58	0.114
**Factor 3—Finding Common Ground**					
Governorate	H(2) = 8.61	0.013	Ramallah vs. Hebron	2.69	0.021
			Ramallah vs. Nablus	2.37	0.035
Origin region	H(2) = 16.55	<0.001	Urban origin vs. Camp origin	−3.32	0.003
			Village origin vs. Camp origin	−2.45	0.015
			Village origin vs. Urban origin	2.67	0.015
Residence type	H(2) = 22.60	<0.001	Urban vs. Camp	−4.16	< 0.001
			Village vs. Camp	−3.20	0.003
			Village vs. Urban	2.71	0.007
Work status	H(5) = 8.88	0.114	…	…	…

Note. * Post Hoc tests used are Dunn–Holm tests.

**Table 8:** HC3-Robust Multiple Regression Predicting Factor 1—Enhancing the Clinician–Patient Relationship (N = 417).

Predictor	*b*	*SE* (HC3)	*p*	95% CI	Std. *β*
Intercept	2.916	0.215	<0.001	[2.493, 3.339]	—
Age	−0.001	0.003	0.851	[−0.006, 0.005]	−0.014
Gender (Male)	−0.002	0.086	0.980	[−0.172, 0.168]	−0.004
Education: Bachelor+	−0.052	0.091	0.570	[−0.230, 0.127]	−0.099
Education: Diploma	−0.133	0.092	0.148	[−0.314, 0.047]	−0.256
Governorate: Hebron	0.088	0.068	0.198	[−0.046, 0.221]	0.169
Governorate: Nablus	0.167	0.082	0.042	[0.006, 0.328]	0.321
Marital: Div/Widowed	0.139	0.137	0.311	[−0.130, 0.409]	0.268
Marital: Single	0.216	0.141	0.126	[−0.061, 0.493]	0.415
Work: Daily worker	−0.079	0.121	0.517	[−0.316, 0.159]	−0.151
Work: Housewife	−0.073	0.128	0.569	[−0.326, 0.179]	−0.141
Work: Public employee	−0.113	0.107	0.289	[−0.323, 0.097]	−0.218
Work: Self-employed	0.081	0.119	0.496	[−0.153, 0.316]	0.156
Work: Unemployed	−0.083	0.122	0.498	[−0.322, 0.157]	−0.159
Origin: Village	−0.129	0.170	0.450	[−0.463, 0.206]	−0.247
Residence: Village	0.139	0.161	0.388	[−0.177, 0.456]	0.268

Note. Model fit: R^2^ = 0.060, adjusted R^2^ = 0.025. Reference categories: Female, Secondary or less, Ramallah, Married, Private employee, Urban/camp. No Type-III omnibus test was significant (all *p* > 0.11).

**Table 9:** HC3-Robust Multiple Regression Predicting Factor 2—Understanding the Whole Person (N = 417).

Predictor	*b*	*SE* (HC3)	*p*	95% *CI*	Std. *β*
Intercept	2.885	0.243	<0.001	[2.407, 3.362]	—
Age	0.000	0.003	0.893	[−0.007, 0.006]	−0.011
Gender (Male)	0.049	0.093	0.598	[−0.133, 0.231]	0.088
Education: Bachelor+	−0.018	0.097	0.854	[−0.208, 0.173]	−0.032
Education: Diploma	−0.137	0.100	0.171	[−0.332, 0.059]	−0.245
Governorate: Hebron	−0.072	0.075	0.337	[−0.220, 0.076]	−0.130
Governorate: Nablus	−0.124	0.086	0.151	[−0.293, 0.045]	−0.222
Marital: Div/Widowed	0.097	0.144	0.500	[−0.186, 0.381]	0.175
Marital: Single	0.202	0.151	0.182	[−0.095, 0.498]	0.362
Work: Daily worker	−0.093	0.134	0.489	[−0.356, 0.171]	−0.166
Work: Housewife	−0.078	0.128	0.540	[−0.330, 0.173]	−0.141
Work: Public employee	−0.114	0.110	0.299	[−0.329, 0.102]	−0.204
Work: Self-employed	−0.007	0.132	0.960	[−0.266, 0.253]	−0.012
Work: Unemployed	−0.034	0.131	0.794	[−0.293, 0.224]	−0.062
Origin: Village	0.031	0.233	0.894	[−0.427, 0.489]	0.055
Residence: Village	0.029	0.229	0.898	[−0.420, 0.479]	0.053

Note. Model fit: R^2^ = 0.031, adjusted R^2^ = −0.005. Reference categories as in Table 7. No Type-III omnibus test was significant (all *p* > 0.31).

**Table 10:** HC3-Robust Multiple Regression Predicting Factor 3—Finding Common Ground (N = 417).

Predictor	*b*	*SE* (HC3)	*p*	95% *CI*	Std. *β*
Intercept	2.861	0.316	<0.001	[2.240, 3.483]	—
Age	0.000	0.004	0.948	[−0.008, 0.009]	0.005
Gender (Male)	0.002	0.111	0.987	[−0.216, 0.219]	0.002
Education: Bachelor+	0.107	0.119	0.370	[−0.127, 0.340]	0.151
Education: Diploma	−0.036	0.111	0.743	[−0.255, 0.182]	−0.051
Governorate: Hebron	−0.085	0.096	0.373	[−0.273, 0.103]	−0.121
Governorate: Nablus	−0.158	0.106	0.136	[−0.365, 0.050]	−0.223
Marital: Div/Widowed	−0.202	0.176	0.250	[−0.548, 0.143]	−0.286
Marital: Single	0.220	0.210	0.296	[−0.193, 0.633]	0.311
Work: Daily worker	−0.166	0.166	0.318	[−0.492, 0.160]	−0.235
Work: Housewife	−0.223	0.155	0.153	[−0.528, 0.083]	−0.315
Work: Public employee	−0.193	0.124	0.121	[−0.438, 0.051]	−0.273
Work: Self-employed	−0.027	0.142	0.848	[−0.307, 0.253]	−0.039
Work: Unemployed	−0.172	0.141	0.222	[−0.449, 0.105]	−0.244
Origin: Village	0.156	0.266	0.557	[−0.367, 0.679]	0.221
Residence: Village	−0.019	0.259	0.941	[−0.529, 0.491]	−0.027

Note. Model fit: R^2^ = 0.058, adjusted R^2^ = 0.023. Reference categories as in Table 7. No Type-III omnibus test was significant (all *p* > 0.31).

## Data Availability

The data presented in this study are available on request from the corresponding author, for privacy reasons.

## Supplementary Materials

The following supporting information can be downloaded at: https://www.mdpi.com/article/10.3390/healthcare14121747/s1, Table S1: Sensitivity Analysis Comparing the Original and Modified PPPC-R Factor Structures.

## Abbreviations

The following abbreviations are used in this manuscript:

PCC	Patient-centred care
PHC	Primary Healthcare
WB	West Bank
DM	Diabetes mellitus
