# Supplementary material for: Listening to the Patient’s Voice: A Quantitative Study on Patient-Centredness in Diabetes Care in Palestinian Public Primary Care Services
Source: Healthcare (Basel). 2026 Jun 17;14(12):1747. doi: 10.3390/healthcare14121747 (PMC13299413; doi:10.3390/healthcare14121747)
Supplement: Supplementary file 1 [file healthcare-14-01747-s001.zip › healthcare-4228399-supplementary.pdf]

Table S1. *Sensitivity Analysis Comparing the Original and Modified PPPC-R Factor Structures*

Panel A. Agreement Between Modified and Original Factor Scores

Factor	*r*	M (modified)	M (original)
F1: Enhancing the Clinician–Patient Relationship	1.00	2.90	2.90
F2: Understanding the Whole Person	.95	2.77	2.68
F3: Finding Common Ground	.90	2.71	2.85

Panel B. Bivariate Omnibus Tests Under the Original Structure

Factor	Predictor	Test	Statistic (df)	*p*
F1	Governorate	Welch ANOVA	F(2, 262.52) = 6.11	.003
F1	Residence type	Welch t	t(302.19) = 0.52	.601
F1	Origin region	Welch t	t(290.75) = 0.68	.495
F2	Governorate	Welch ANOVA	F(2, 259.39) = 0.14	.867
F2	Residence type	Welch t	t(277.83) = −0.74	.460
F2	Origin region	Welch t	t(282.40) = −0.77	.441
F3	Governorate	Welch ANOVA	F(2, 246.72) = 11.61	< .001
F3	Residence type	Welch t	t(317.84) = −3.05	.002
F3	Origin region	Welch t	t(308.36) = −2.90	.004

Panel C. Multivariable Model Fit Under the Original Structure

Factor	R^2	*Adjusted* R^2	*n*
F1: Enhancing the Clinician–Patient Relationship	.060	.025	417
F2: Understanding the Whole Person	.023	−.014	417
F3: Finding Common Ground	.074	.039	417

Panel D. Multivariable HC3-Robust Type III Omnibus Tests (p Values) Under the Original Structure

Predictor	F1	F2	F3
Age	.851	.847	.958
Gender	.980	.326	.608
Education level	.348	.168	.314
Governorate	.117	.830	.052
Marital status	.166	.419	.301
Work status	.476	.980	.374
Origin region	.450	.902	.696
Residence type	.388	.978	.886

Note. Factor scores were recomputed using the original three-domain PPPC-R assignment, in which the two treatment-related items (“explain treatment” and “explore manageability”) were retained in Finding Common Ground rather than reassigned to Understanding the Whole Person. F1 = Enhancing the Clinician–Patient Relationship; F2 = Understanding the Whole Person; F3 = Finding Common Ground. In Panels B and D, residence type and origin region were collapsed to two categories (camp combined with urban as the reference). Welch t and Welch ANOVA used Satterthwaite-adjusted degrees of freedom. Type III omnibus tests used HC3 heteroskedasticity-consistent standard errors. No omnibus test reached significance after adjustment (all $p > .05$). N = 417.
